# Truncated SSX Protein Suppresses Synovial Sarcoma Cell Proliferation by Inhibiting the Localization of SS18-SSX Fusion Protein

**DOI:** 10.1371/journal.pone.0077564

**Published:** 2013-10-09

**Authors:** Yasushi Yoneda, Sachio Ito, Toshiyuki Kunisada, Yuki Morimoto, Hirotaka Kanzaki, Aki Yoshida, Kenji Shimizu, Toshifumi Ozaki, Mamoru Ouchida

**Affiliations:** 1 Department of Orthopedic Surgery, Graduate School of Medicine, Dentistry and Pharmaceutical Sciences, Okayama University, Okayama, Japan; 2 Department of Molecular Genetics, Graduate School of Medicine, Dentistry and Pharmaceutical Sciences, Okayama University, Okayama, Japan; 3 Department of Medical Materials for Musculoskeletal Reconstruction, Graduate School of Medicine, Dentistry and Pharmaceutical Sciences, Okayama University, Okayama, Japan; INRS, Canada

## Abstract

Synovial sarcoma is a relatively rare high-grade soft tissue sarcoma that often develops in the limbs of young people and induces the lung and the lymph node metastasis resulting in poor prognosis. In patients with synovial sarcoma, specific chromosomal translocation of t(X; 18) (p11.2;q11.2) is observed, and SS18-SSX fusion protein expressed by this translocation is reported to be associated with pathogenesis. However, role of the fusion protein in the pathogenesis of synovial sarcoma has not yet been completely clarified. In this study, we focused on the localization patterns of SS18-SSX fusion protein. We constructed expression plasmids coding for the full length SS18-SSX, the truncated SS18 moiety (tSS18) and the truncated SSX moiety (tSSX) of SS18-SSX, tagged with fluorescent proteins. These plasmids were transfected in synovial sarcoma SYO-1 cells and we observed the expression of these proteins using a fluorescence microscope. The SS18-SSX fusion protein showed a characteristic speckle pattern in the nucleus. However, when SS18-SSX was co-expressed with tSSX, localization of SS18-SSX changed from speckle patterns to the diffused pattern similar to the localization pattern of tSSX and SSX. Furthermore, cell proliferation and colony formation of synovial sarcoma SYO-1 and YaFuSS cells were suppressed by exogenous tSSX expression. Our results suggest that the characteristic speckle localization pattern of SS18-SSX is strongly involved in the tumorigenesis through the SSX moiety of the SS18-SSX fusion protein. These findings could be applied to further understand the pathogenic mechanisms, and towards the development of molecular targeting approach for synovial sarcoma.

## Introduction

Synovial sarcoma is a relatively rare high-grade soft tissue sarcoma that often develops in the limbs of young people. Recent advancements in surgery, chemotherapy, radiotherapy, and multidisciplinary therapy have improved the prognosis. The overall 5-year survival rate in synovial sarcoma patients without metastasis is reported to be 61–80% [1-4]. However, in long-term, synovial sarcoma cells sometimes metastasize to the lung and the lymph node, turning into a life-threatening condition resulting in a poor prognosis. The pathogenic mechanisms have not yet been completely elucidated. However, specific chromosomal translocation of t(X; 18) (p11.2;q11.2) has been identified in patients with synovial sarcoma [5]. The fusion of the *SS18* gene on chromosome 18 to the *SSX* gene on chromosome X results in the expression of fusion protein SS18-SSX composed of the NH_3_-terminal half of the amino acids from SS18 and COOH-terminal amino acids of the SSX. Expression of the fusion protein has been observed in more than 97% of synovial sarcoma cells [6-8]. This suggests that the SS18-SSX fusion protein is specifically expressed in the synovial sarcoma cells, and is important in the pathogenesis of the disease. SS18 and SSX proteins are localized in the nucleus and are associated with transcriptional regulation, although neither have a distinct DNA binding-domain [6-8]. Therefore, they are thought to regulate transcription by interacting with other proteins that can bind directly to the DNA in the nucleus.

The wild-type SS18 protein comprises 387 amino acid residues, being ubiquitously expressed in normal cells [6]. It has been reported that SS18 interacts with SNF/SWI complexes (a chromatin remodeling factor) [9-13], Sin3A (a factor of histone deacetylase complex) [14,15], p300 [16], and AF10 [17]. SS18 is regarded as a transcriptional co-activator because it promotes transcription.

Wild-type SSX protein consists of 188 amino acid residues [7], and it is expressed in the testis and the thyroid, along with melanoma and lung cancer tumor cells, and is one of the cancer/testis antigens [18-21]. Major fusion partners of *SS18* in synovial sarcoma are *SSX1* and *SSX2*, and *SSX4* has been reported in rare cases [22,23]. The *SSX1* to *SSX9* genes have been identified [24]. SSX is reported to interact with transcriptional repressors such as the polycomb-group (PcG) [25-29], core histone [11], RAB3IP, and SSX2IP [27,28,30], and is regarded as a transcriptional co-repressor since it suppresses transcription.

SS18-SSX fusion protein is also known to be localized in the nucleus [25,26,31,32], and is reported to interact with a variety of proteins [10-12,25,26,29,33]. Recently, gene expression profiles using DNA microarray has revealed various downstream genes that are targeted by the SS18-SSX fusion protein [34-42]. The control of gene expression by SS18-SSX is believed to involve chromatin remodelingbecause of SS18-SSX’s colocalization with both Trithorax (TrxG) and Polycomb group (PcG) complexes, thereby maintaining chromatin in a poised bivalent state [26,39,43]. Lubieniecka et al. reported that *EGR1* is repressed by the SS18-SSX protein through trimethylation of histone H3, and HDAC inhibitor reverses the histone modifications and reactivates *EGR1* expression in synovial sarcoma cells [43]. Su et al. identified ATF2 as the DNA-binding partner of SS18-SSX and showed that HDAC inhibitors reverse the epigenetic repressor activity of the SS18-SSX oncoprotein complex by preventing TLE1 recruitment[44]. . Several studies have showed that synovial sarcoma cells express mRNA transcripts of pluripotency factors such as *Sox2*, *Oct3/4*, and *Nanog* [45] and show stem-cell-like gene expression profiles [46], and that tumor cells lacking the BAF47 tumor suppressor subunit express stem-cell-like signatures [47]. Kadoch and Crabtree [48] demonstrated that SS18-SSX fusion protein binds to SWI/SNF-like BAF (chromatin-remodeling) complexes and evicts both the wild-type SS18 and the tumor suppressor BAF47. This altered complex binds to and activates the Sox2 locus by disrupting H3K27me3-mediated repression, and drives proliferation of these cells [48]. In transgenic mice, conditional overexpression of SS18-SSX2 in the myogenic progenitor compartment, but not that in other compartments, leads to the appearance of both monophasic and biphasic synovial sarcoma tumors with full penetrance [49].

Generally, proteins that function as transcriptional factors are believed to form complicated complexes, localize at specific region, and carry out their own functions. Synovial sarcoma cell line SYO-1 bearing the *SS18-SSX2* translocation was established previously [50]. We investigated the localization pattern of each component of synovial sarcoma-related fusion protein, and examined the inhibiting effect of the localization of SS18-SSX protein in order to understand the mechanisms by which SS18-SSX contributes towards the synovial sarcoma pathogenesis.

## Results

### Localization patterns of synovial sarcoma-related proteins in the SYO-1 cells

**Figure 1:**
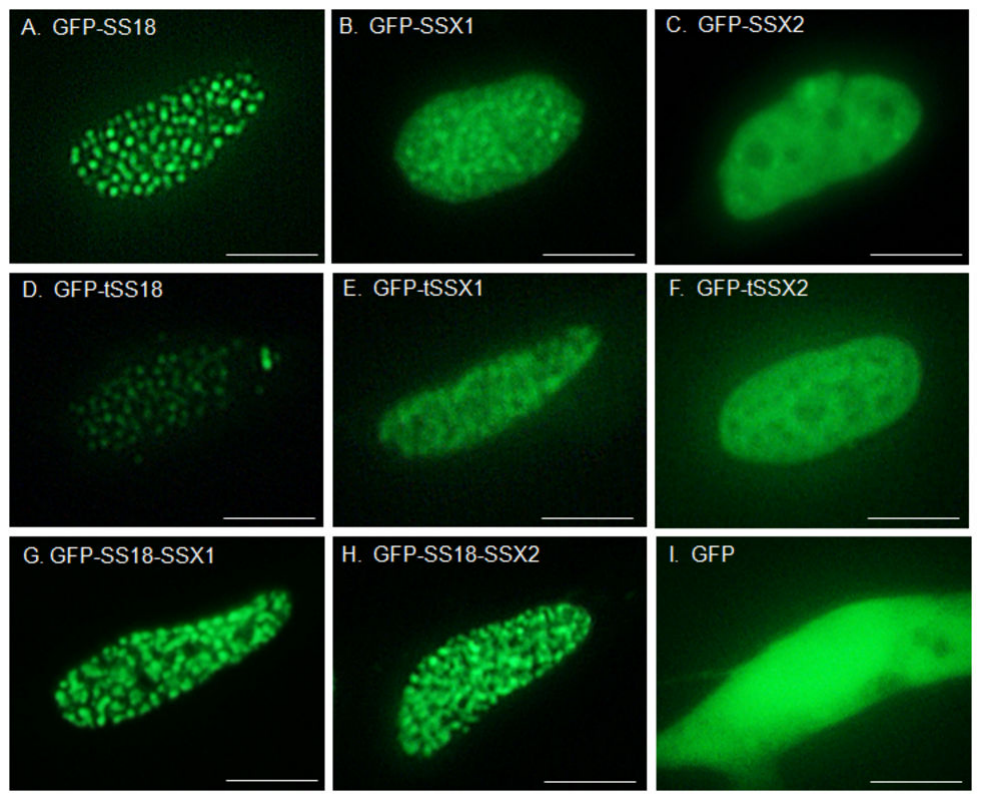
Localization of synovial sarcoma-related proteins in synovial sarcoma SYO-1 cells. GFP fused proteins were observed using a fluorescence microscope. A, GFP-SS18; B, GFP-SSX1; C, GFP-SSX2; D, GFP-tSS18; E, GFP-tSSX1; F, GFP-tSSX2; G, GFP-SS18-SSX1; H, GFP-SS18-SSX2; I, GFP. Scale bars indicate 5 µm.

Localization of full-length SS18 and SSX proteins tagged to GFP was observed under fluorescence microscope, after the constructs pEGFP-*SS18*, pEGFP-*SSX1*, and pEGFP-*SSX2* were transfected into SYO-1 cells. SS18 localized to the nucleus and showed a speckled distribution pattern (Figure 1A). Both SSX1 and SSX2 localized in the nucleus and displayed a diffuse localization pattern. SSX1 also displayed a speckled pattern and the number of these speckles were relatively more in cells transfected with SSX1 than (Figure 1B) compared to cells with SSX2 (Figure 1C). Next, SS18-SSX fusion proteins were observed after transfection of pEGFP-*SS18-SSX1* and pEGFP-*SS18-SSX2* into SYO-1 cells. Both SS18-SSX1 and SS18-SSX2 were localized in the nucleus showing clear speckles similar to that observed for SS18. However, when we examined closely, compared to SS18, the fusion proteins displayed a pattern in which densely packed oval dots were more evenly distributed (Figure 1G and 1H). There was no remarkable difference in the localization between SS18-SSX1 and SS18-SSX2. To examine the effect of the fluorescent protein on the gene localization, HEK293 cells transfected with SS18, SSX2, and SS18-SSX2 without GFP were analyzed by fluorescence immunocytochemistry with anti-SS18 and anti-SSX antibodies. SS18 and SS18-SSX2 staining showed a speckled pattern and SSX2 displayed a diffuse pattern, which is similar to the localization of the GFP-tagged proteins (Figure S1), suggesting that addition of the fusion protein does not affect gene localization. Then, the GFP-fusion plasmids harboring *SS18* moiety and *SSX* moiety were constructed. Localization of tSS18 (truncated SS18 composing of 1-379 amino acids), tSSX1, and tSSX2 (truncated SSX composing of 111-188 amino acids) was observed after transfection of pEGFP-*tSS18*, pEGFP-*tSSX1* and pEGFP-*tSSX2* into SYO-1 cells. The tSS18 showed a similar localization pattern in the nucleus as that of SS18, although the intensity of the fluorescence was weak (Figure 1D). Localization pattern of tSSX1 and tSSX2 did not differ remarkably from those of SSX1 and SSX2, respectively (Figure 1E and F). We also looked at the localization patterns of these synovial sarcoma-related proteins in HEK293 cells transfected with these plasmids, the localization patterns were similar to that observed in SYO-1 cells (Figure S2). We confirmed that the proteins from these plasmid constructs were successfully expressed in HEK293 cells by western blotting with anti-SS18 and anti-SSX antibodies (Figure S3).

### Change in the localization pattern of SS18-SSX upon co-expression with truncated SS18 and SSX proteins

**Figure 2:**
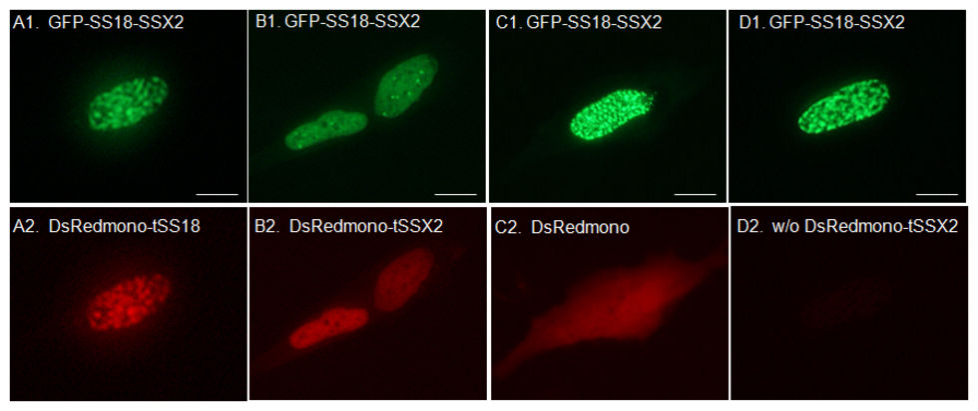
Representative images showing the co-expression of GFP-tagged SS18-SSX2 and of DsRed-monomer-tagged truncated proteins in transfected SYO-1 cells. Fluorescent proteins were observed using a fluorescence microscope. A, co-expression of GFP-SS18-SSX2 (A1) and DsRed-monomer-tSS18 (A2); B, co-expression of GFP-SS18-SSX2 (B1) and DsRed-monomer-tSSX2 (B2); C, co-expression of GFP-SS18-SSX2 (C1) and DsRed-monomer empty vector (C2); D, co-transfection of GFP-SS18-SSX2 (D1) and DsRed-monomer-tSSX2 (D2) SYO-1 cell in which tSSX2 was not expressed after co-transfection. Scale bars indicate 5 µm.

**Figure 3:**
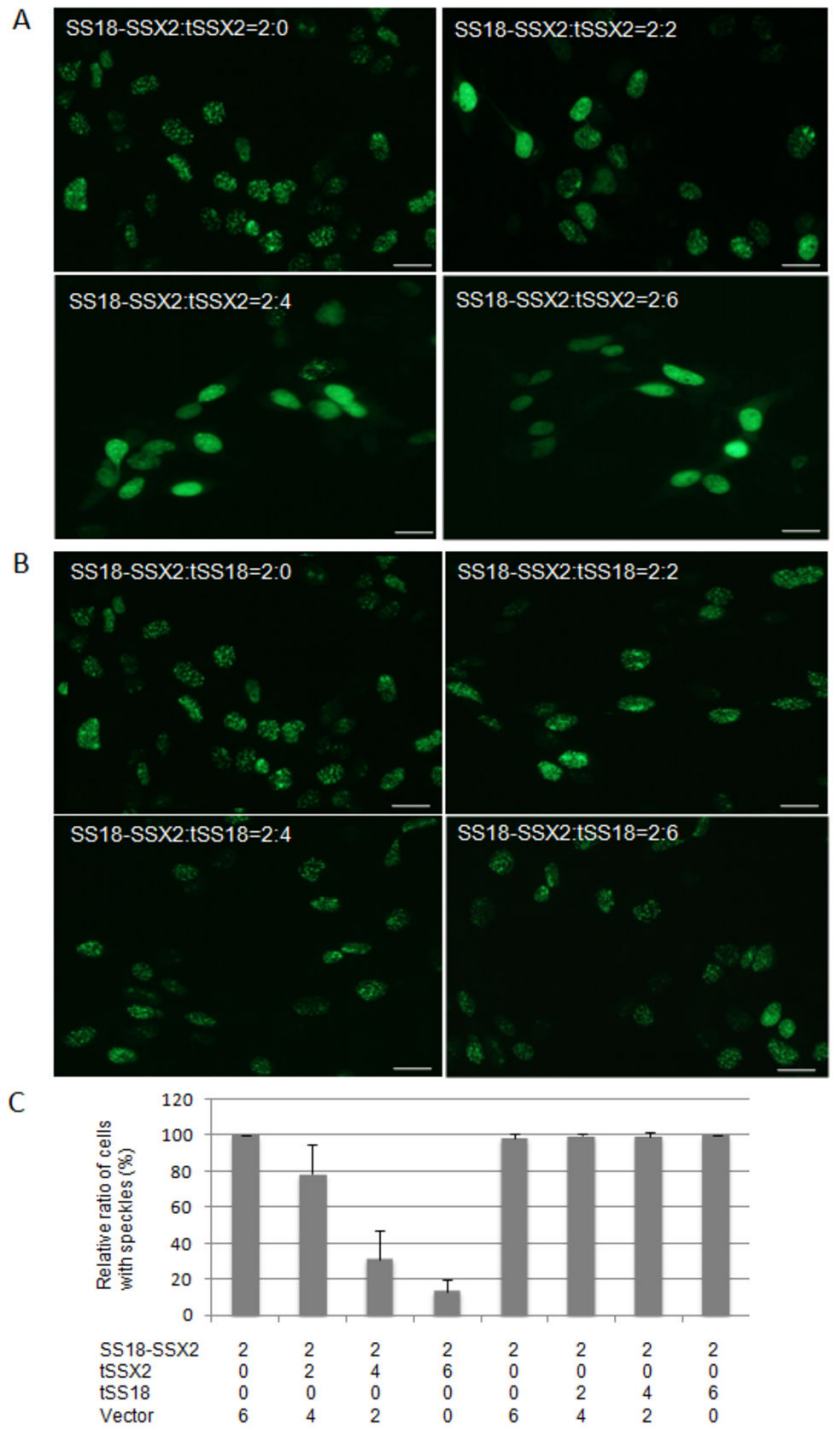
Effect of increasing expression of tSSX on the localization of SS18-SSX. Plasmid pEGFP-SS18-SSX2 (2 µg) was co-transfected in HEK293 cells with pDsRedmonomer-*tSSX2* (0, 2, 4, and 6 µg) (A), or DsRedmonomer-*tSS18* (0, 2, 4, and 6 µg) (B). The total DNA amount of transfection was complimented by pCMV-Tag2B empty plasmid without fluorescent protein (6, 4, 2, and 0 µg). The cells showing either a speckled pattern or a diffuse pattern of SS18-SSX2 localization were counted in 30 fields of fluorescence microscope, and the relative ratio of cells with speckled pattern was calculated (C). Scale bars indicate 10 µm.

Effect of tSS18 and tSSX truncated proteins on localization of SS18-SSX was examined in synovial sarcoma cells. When GFP-tagged *SS18-SSX2* was co-transfected along with DsRedmonomer-*tSS18* into SYO-1 cells, the localization pattern of SS18-SSX2 was not remarkably different from that observed in cells that were transfected with SS18-SSX2 alone, but the localization of tSS18 was similar with that of SS18-SSX2 (Figure 2, A1, and A2). We then looked at the changes in the localization pattern if any of the GFP-tagged *SS18-SSX2* when co-expressed with DsRedmonomer-*tSSX2* fusion in SYO-1 cells. The localization of SS18-SSX2 significantly changed from a speckled pattern to a diffuse localization pattern, and was similar to that of tSSX2 (Figure 2, B1, and B2). When GFP-tagged *SS18-SSX2* and DsRedmonomer-*tSSX1* fusion were co-expressed, localization of SS18-SSX2 also changed from speckled to a diffuse pattern, and was similar to that of tSSX1 (data not shown). When *SS18-SSX1* was co-expressed with *tSSX1*, the localization of SS18-SSX1 also showed to a diffuse pattern (Figure S4). However, when pEGFP-*SS18-SSX2* and pDsRedmonomer empty vector were co-expressed, localization of SS18-SSX2 did not change (Figure 2, C1 and C2). When we transfected SYO-1 cells with DsRedmonomer-fused *SS18*, *SSX1* and *SSX2* full-length genes instead of using the truncated genes, the localization of SS18-SSX was similar to the localization pattern observed earlier (data not shown). We obtained similar results for the localization of these fusion proteins in HEK293 cells transfected with the above plasmids (Figures S5 and S6). All the cells displaying a diffuse pattern we examined were observed to have red-fluorescence of tSSX2 in their nuclei. Therefore, we analyzed quantitatively the effect of increasing expression of tSSX on localization of SS18-SSX. Transfection of increasing amount of DsRedmonomer-*tSSX2* plasmid (0, 2, 4, and 6 µg) showed significant loss of cells with speckled pattern. However, when cells were transfected with increasing amounts of DsRedmonomer-*tSS18* plasmid, there was no change in the localization pattern (Figure 3). We examined the effect of DsRedmonomer-tSSX2 and -tSS18 on GFP-SS18-SSX2 expression in transfected HEK293 cells by western blotting analysis. As can be seen in the Figure S7, expression of SS18-SSX2 was not affected by tSSX2 and tSS18.

### Suppression of cell proliferation of SYO-1 cells by exogenous expression of tSSX2

**Figure 4:**
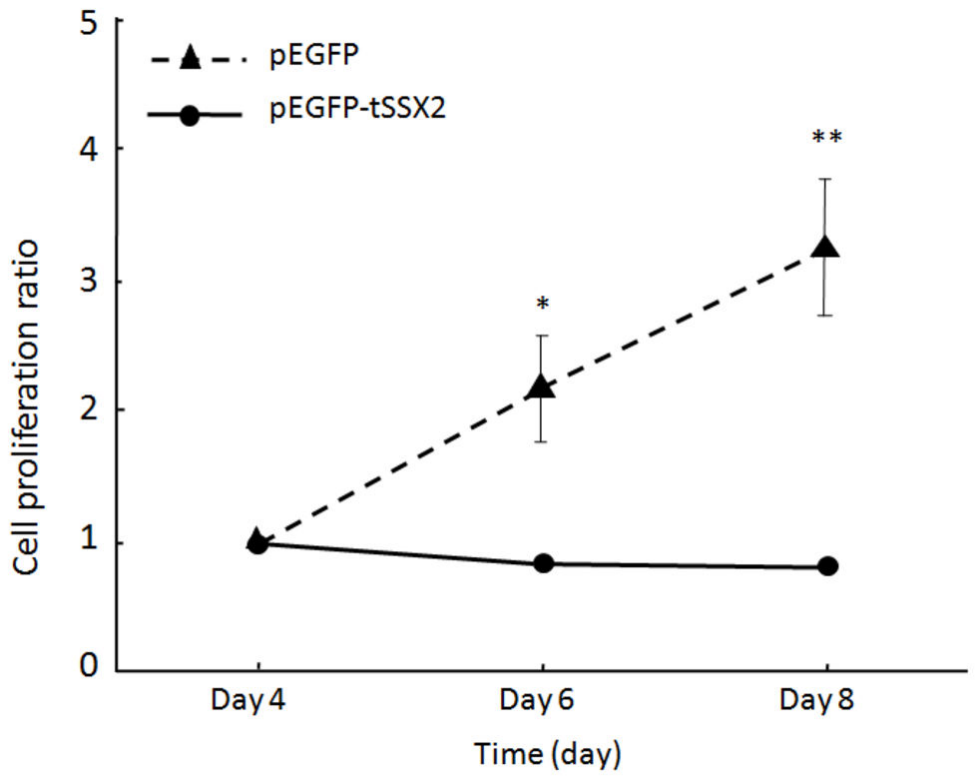
Exogenous expression of tSSX2 suppresses the proliferation of synovial sarcoma SYO-1 cells. SYO-1 cells were transfected with pEGFP-*tSSX2* or pEGFP vector, split 48 h after transfection, and the cells expressing the GFP-tagged fusion proteins were counted on day 4, 6 and 8 after transfection. The cell proliferation ratio was normalized by dividing the number of cells expressing GFP-tagged proteins on days 6 and 8 by the number on day 4. Error bars indicate standard deviation, *p < 0.001, **p < 0.0001.

**Figure 5:**
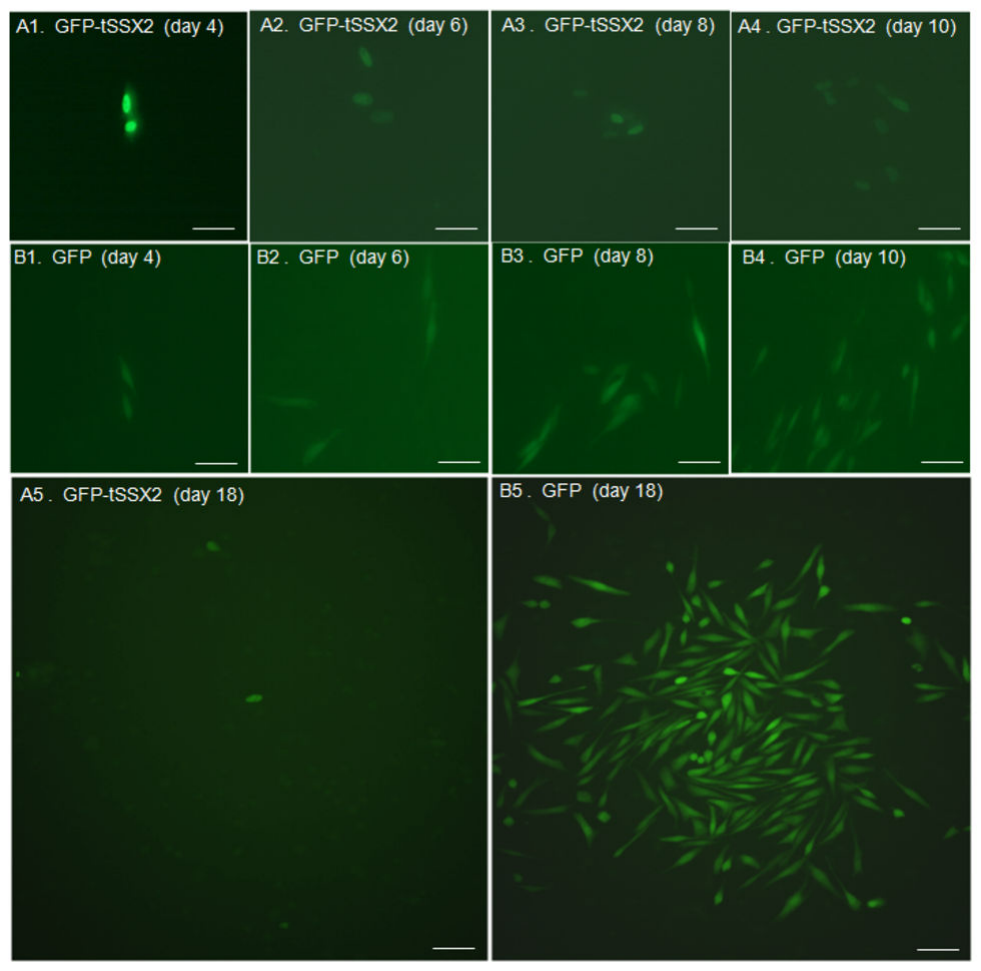
Representative images showing changes in proliferation of SYO-1 cells expressing GFP-*tSSX2* monitored for a period of 18 days. SYO-1 cells were transfected with pEGFP-*tSSX2* (A) or pEGFP vector (B), split 48 h after transfection, and the cells expressing GFP-tagged proteins were observed under fluorescence microscope on day 4, 6, 8, 10, and 18 after transfection. 1, day 4; 2, day 6; 3, day 8; 4, day 10; 5, day18. Scale bars indicate 20 µm.

As described above, the localization of SS18-SSX2 was affected by the co-expression of tSSX2 in SYO-1 cells. Therefore, we examined the effect of expression of *tSSX2* on proliferation of SYO-1 cells harboring the *SS18-SSX2* fusion gene. SYO-1 cells were transfected with GFP-fused *tSSX2* or GFP vector alone as control, split 48 h after transfection, and the cells expressing the fluorescence proteins were observed and counted on day 4, 6, and 8 after transfection. The cell proliferation ratio was normalized by dividing the numbers of GFP expressing cells on days 6 and 8 by the number on day 4 in SYO-1 cells transfected with *tSSX2* and control group. The cell proliferation was significantly suppressed in the tSSX2 group on days 6 and 8 (Figure 4). A time-course experiment showing the change in the number of GFP expressing cells is shown in Figure 5.

### Suppression of colony formation of synovial sarcoma cells by exogenous expression of tSSX

**Figure 6:**
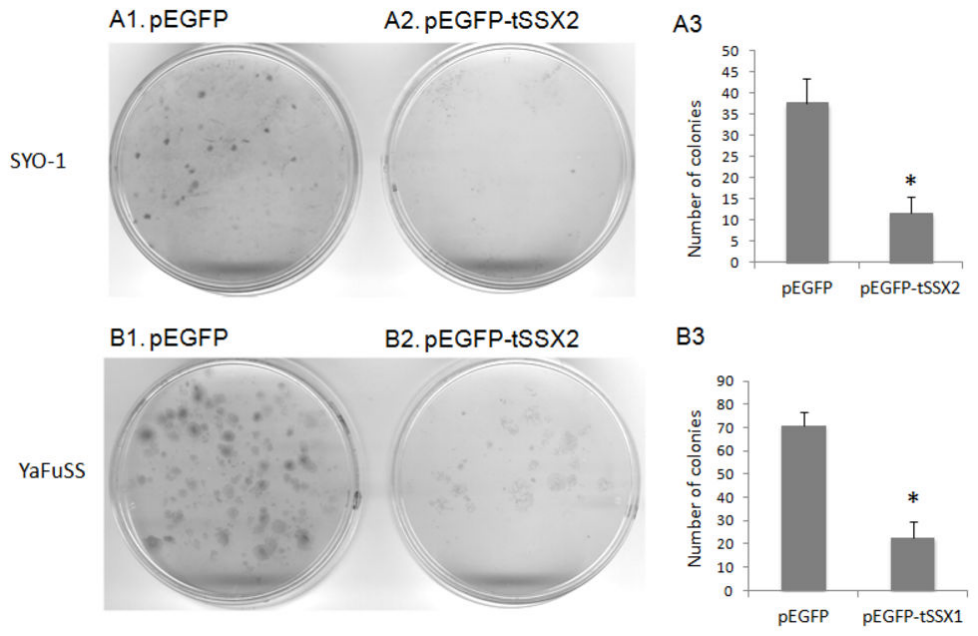
A. Exogenous expression of tSSX suppresses the colony formation in SYO-1 cells. SYO-1 cells cultured in 90 mm dishes were transfected with pEGFP empty vector (A1) or pEGFP-*tSSX2* (A2), split into 10 plates of 60 mm dishes 48 h after transfection and selected with G418 for three weeks. The cells were fixed in 4% formaldehyde and stained with Giemsastain solution. B, YaFuSS cells were transfected with pEGFP empty vector (B1) or pEGFP-*tSSX2* (B2), and assessed for colony formation activity. The colonies were counted and the average number of colonies formed is shown as bar graphs for SYO-1 (A3) and YaFuSS (B3). Error bars indicate standard deviation, * *p* < 0.01.

We examined the effect of *tSSX2* expression on colony formation of SYO-1 cell line. SYO-1 cells were transfected with GFP-tagged *tSSX2* or GFP vector alone, split 48 h after transfection, and selected with G418 for three weeks. The colonies were observed after cell fixation and staining. The number of colonies formed as well as the size of the colony was decreased in the *tSSX2* group as compared with that of the control group (Figure 6A). We also studied the colony forming ability of another synovial sarcoma cell line YaFuSS harboring the *SS18-SSX1* fusion gene. Reduced number of colonies was observed in YaFuSS cell line transfected with GFP-tagged *tSSX1* (Figure 6B).

## Discussion

In the present study, the wild-type SS18 clearly showed a speckled localization pattern, while SSX showed primarily a diffuse pattern. The localization pattern of SS18-SSX fusion proteins was clearly nuclear with a speckled pattern and clear elliptical dots were densely distributed, which differed from those of the wild-type SS18 and SSX. In addition, localization of tSS18 and tSSX, which are components of SS18-SSX fusion protein did not remarkably differ from that of the wild-type SS18 and SSX, respectively. Hence, the localization pattern of SS18-SSX is thought to be the distinct feature obtained only when both SS18 and SSX form a fusion protein. The subtle difference of tSSX1 and tSSX2 localization is considered to depend on 11 different amino acids between them.

Several investigators have reported regarding the localization of synovial sarcoma-associated proteins. dos Santos et al. have reported that the SS18 displays a nuclear punctated localization pattern and SSX a diffuse pattern in the nucleus of transfected COS-1 cells [31]. They suggested that SS18 might influence the manifestation of the tumor since the SS18-SSX fusion protein is also localized in the nucleus and displays a similar punctated pattern [31]. Brett et al. also reported that SS18 and SSX1 displayed a speckled and uniform distribution pattern in the nucleus, respectively, and that the localization pattern of SS18-SSX2 fusion protein is similar to that of SS18 in transfected NIH3T3, Cos-7, HT1080, and MRC-5 cells [32]. On the other hand, Soulez et al. reported that the co-localization of SS18-SSX fusion protein and SSX with RING1 and BMI1, which belong to polycomb group (PcG), but not SS18 [26]. dos Santos et al. subsequently reported that HeLa and COS-1 cells harboring the *SSX* expression vector displayed speckles in the diffuse distribution, and the localization of speckles of SS18-SSX coincided with that of SSX [25]. Furthermore, when the C-terminus of the SSX region called the SSX repression domain was removed, the localization of SS18-SSX coincided with that of SS18 [26,51]. Therefore, they concluded that SSX region played a dominant role over SS18 region in localization of SS18-SSX and that the C-terminus of SSX was especially important [25].

In our study, we demonstrate that the localization pattern of SS18-SSX changes significantly when co-expressed with tSSX, suggesting that the localization of SS18-SSX can be antagonized at least by tSSX. These results indicate that SS18-SSX might bind to other proteins via its SSX region; this agrees well with the results of Soulez et al. and dos Santos et al. [25,26]. As the localization of SS18-SSX changed to a diffuse pattern upon co-expression of tSSX and this seems to coincide with the localization pattern of SSX and tSSX, the localization of SS18-SSX might be guided through the SSX region of SS18-SSX. Interestingly, since co-expression of tSSX2 suppressed cell proliferation and colony formation of the synovial sarcoma SYO-1 and YaFuSS cell lines, the speckle distribution pattern characterized by SS18-SSX might be strongly involved in tumorigenesis of synovial sarcoma cells. Recently, Kadoch and Crabtree [48] demonstrated that SS18-SSX protein binds to SWI/SNF-like BAF (chromatin-remodeling) complexes, and that SS18-SSX-driven altered BAF complex formation depends on 2 amino acids of SSX [48]. Our results showing disappearance of SS18-SSX speckles by exogenous tSSX transfection agrees with their results, and the phenomenon we found might show the disruption of SS18-SSX-driven altered BAF complex antagonized by tSSX. The effect of tSSX on SS18-SSX speckle disruption might depend on 2 amino acids of SSX at positions 43 and 44. The authors also demonstrated that assembly of wild-type complexes and proliferative quiescence can be achieved by increasing the concentration of wild-type SS18. However, we have not performed a cell growth assay using tSS18 transfection because we could not find any change of SS18-SSX localization by tSS18 transfection due to similarity of localization of SS18-SSX and tSS18. Our finding that tSS18 and SS18 colocalize with SS18-SSX spatially in the nucleus might explain the results that increased expression of SS18 displaces SS18-SSX from SWI/SNF-like BAF complexes and lead to reduced growth. Perani et al. reported that SS18 forms an oligomer with SS18 itself or with SS18-SSX [9]. If SS18-SSX forms an oligomer with tSS18, it could account for the same localization pattern observed for SS18-SSX and tSS18.

SSX1 and SSX2 interact with BMI1 and RING1A, which belong to PcG and with LHX4, RAB3IP, and SSX2IP which are transcription factors [27,28]. RAB3IP and SSX2IP interact with the N-terminal domain of SSX [27,30]. Since SS18-SSX fusion proteins do not consist of the interaction domains, RAB3IP and SSX2IP are quite unlikely to be the candidate proteins interacting with SS18-SSX. Our results using SSX were similar between the two subtypes of SSX, and it is known that PcGs such as BMI1 and RING1A interact with SSX1 and SSX2 commonly. Therefore, BMI1 and RING1A could be the candidate proteins interacting with the SSX1 or SSX2 region of SS18-SSX fusion protein.

Our results revealed the possibility that SS18-SSX is involved in tumor proliferation because of its interaction with some specific proteins interacting with the wild-type SSX via the SSX region of SS18-SSX. Further study is needed to identify these interacting proteins, which will provide a better understanding on the pathways involved in the pathogenesis of synovial sarcoma. This could provide new target molecules that could help in the development of newer treatment options for synovial sarcoma using molecular targeting approach.

## Materials and Methods

### Cell lines

Human synovial sarcoma cell line SYO-1 expressing the *SS18-SSX2* fusion gene was established in our laboratory [50]. Human synovial sarcoma cell line YaFuSS expressing the *SS18-SSX1* fusion gene was kindly provided by Dr. J. Toguchida (Institute for Frontier Medical Sciences, Kyoto University, Japan) [52]. Human normal embryonic kidney cell line HEK293 was purchased from American Type Culture Collection. These cell lines were grown in Dulbecco’s modified Eagle’s medium or RPMI-1640 (Invitrogen, Carlsbad, CA, USA) supplemented with 10% fetal bovine serum (Invitrogen), 100 units/ml of penicillin G and 100 µg/ml of streptomycin (Meiji Seika, Tokyo, Japan). All cells were incubated at 37°C in a humidified atmosphere containing 5% CO_2_.

### Plasmid construction

The coding regions of the human *SS18-SSX1*, *SS18-SSX2*, *SS18*, *SSX1*, *SSX2*, *tSS18* (truncated *SS18* coding #1-379 amino acids), *tSSX1* and *tSSX2* (truncated *SSX* coding #111-188 amino acids) were amplified by PCR with cDNA derived from synovial sarcoma cells as described earlier [14]. The amplified cDNAs were inserted downstream of green fluorescent protein (GFP) of pEGFP-C vector (Clontech), and the expression plasmids were constructed to produce GFP tagged-*SS18-SSX1*, -*SS18-SSX2*, *-SS18*, *-SSX1*, *-SSX2*, *-tSS18*, *-tSSX1* and *-tSSX2* proteins. Furthermore, pDsRedmonomer plasmids bearing the *SS18*, *SSX1*, *SSX2*, *tSS18*, *tSSX1* and *tSSX2* cDNAs were inserted downstream of DsRedmonomer, to produce DsRedmonomer fusion proteins. To detect the localization of SS18, SSX2, and SS18-SSX2 by fluorescence immunocytochemistry, pCMV-Tag2B expression plasmids with FLAG tag containing the cDNAs were used for transfection as described [53]

### Transfection

To increase the transfection efficiency, reverse transfection method was used. The vectors (total DNA amount of 0.25 µg in cases where one kind of vector was used, and 0.125 µg each in case of two kinds of vectors) were mixed with 1 µL of Effectene (QIAGEN), 3.25 µL EC-buffer, 1 µL enhancer and 0.6 µL of 1.5 M sucrose; 9 µL of gelatin was added 15 min later, and the mixture was dropped into a well (12 mm × 10 mm) of a 8 well tissue culture chamber slide (Lab-Tec, Nunc) and allowed to dry. The cells were plated in the wells, and the localization of fluorescent proteins was observed under fluorescence microscope after replacement of the medium with DMEM without phenol red.

### Fluorescence immunocytochemistry

To determine the localization of SS18 and SSX in transfected HEK293 cells, the cells were seeded on glass culture slides (BD Falcon 8-well CultureSlide; BD Biosciences), and grown to 80% confluence. They were then fixed in 1% formaldehyde for 10 min at room temperature, permeabilized, blocked with 1% bovine serum albumin (BSA) in PBS for 30 min at room temperature, and then incubated with anti-SS18 antibody (SYT; C-19, Santa Cruz Biotechnology, California, USA) or anti-SSX1 antibody (FL-188, Santa Cruz Biotechnology) overnight. The cells were washed and then incubated simultaneously with Alexa 594-conjugated secondary antibody (Invitrogen, Eugene, OR) for 1 h and Hoechst 33342 (1 mg/mL) (ICN Biomedicals, Aurora, OH) for nuclear staining. Images were acquired with SenSys0401E (Roper Scientific Germany, Ottobrunn, Germany), DMRA2 (Leica Microsystems, Wetzlar, Germany) and Leica Cytogenetic Workstation (CW4000; Leica Microsystems Imaging Ltd, Cambridge, UK).

### Western blotting analysis

The protein samples (10 µg total proteins) were combined with gel-loading buffer, heated to 95°C for 10 min, and then separated on 12% polyacrylamide gels. The proteins were subsequently transferred onto PVDF membranes (Invitrogen) and blocked overnight at 4°C in 3% BSA/PBS. The membranes were incubated at room temperature with anti-SS18 antibody for 4 h (SYT; H-80, Santa Cruz Biotechnology) or anti-SSX1 antibody (FL-188, Santa Cruz Biotechnology). β-actin (Sigma, Saint Louis, USA) was used as a loading control. After washing with PBS/0.05% Tween-20, the filters were incubated with alkaline phosphatase-conjugated antibodies. The protein signal was visualized using FLA-3000 (Fujifilm).

### Cell proliferation assay

The plasmid pEGFP (control group) or pEGFP-*tSSX2* (tSSX2 group) was transfected into SYO-1 cells cultured in 60 mm culture dish, and split into 4 plates of 60 mm cell culture dishes 48 h after transfection. The transfectants expressing the fluorescent proteins were observed and counted under a fluorescence microscope 4, 6 and 8 days after transfection. Ratio of the number of cells on days 6 and 8 to that of day 4 was calculated, and compared between tSSX2 and the control group.

### Colony formation assay

SYO-1 cells were grown in 90 mm culture dish and were transfected with the plasmid pEGFP (control group) or pEGFP-*tSSX2* (tSSX2 group), split into 10 plates of 60 mm culture dishes 48 h after transfection, selected with 400 µg/mL of G418 for two weeks, and stained with Giemsa stain solution after 4% formaldehyde fixation.

### Statistical analysis

Comparison between the two groups in the cell proliferation assay was performed using *t*-test and *p* < 0.05 were considered statistically significant. StatView version 5.0 (SAS Institute Inc., Cary, North Carolina) was used for statistical analysis.

## Supporting Information

Figure S1 **Localization of synovial sarcoma-related proteins by fluorescence immunocytochemistry.** HEK293 cells were transfected with pCMV-Tag2B-SS18, pCMV-Tag2B-SSX2, and pCMV-Tag2B-SS18-SSX2, and analyzed by fluorescence immunocytochemistry with anti-SS18 and anti-SSX antibodies. The transfected cells with SS18 and SS18-SSX2 were reacted with anti-SS18 antibody (upper and middle, respectively), and the SSX2 transfectant was reacted with anti-SSX antibody (lower). Left, antibody reaction using Alexa 594-conjugated secondary antibody; middle, Hoechst33342 staining; right, merged image. The scale bars are 5-µm long.
(TIF)

Figure S2 **Localization of synovial sarcoma-related fusion proteins in HEK293 cells.** Cells expressing GFP-tagged proteins were observed under a fluorescence microscope. A, GFP-SS18; B, GFP-SSX1; C, GFP-SSX2; D, GFP-SS18-SSX1; E, GFP-SS18-SSX2; F, GFP. Scale bars indicate 5 µm.
(TIF)

Figure S3 **Confirmation of expression of recombinant proteins by Western blotting.** A: HEK293 cells were transfected with pEGFP (lane 1); pEGFP-SS18 (lane 2, about 79 kDa); pEGFP-SS18-SSX1 (lane 3, about 83 kDa); pEGFP-SS18-SSX2 (lane 4, about 83 kDa); pEGFP-tSS18 (lane 5, about 77 kDa); pDsRedmonomer-tSS18 (lane 6, about 81 kDa); and pDsRedmonomer (lane 7); and the cell extracts were detected by western blotting with anti-SS18 antibody. B: HEK293 cells were transfected with pEGFP (lane 1); pEGFP-SSX1 (lane 2, about 51 kDa); pEGFP-tSSX1 (lane 3, about 38 kDa); pDsRedmonomer-tSSX1 (lane 4, about 42 kDa); pDsRedmonomer (lane 5); pEGFP (lane 6); pEGFP-SSX2 (lane 7, about 51 kDa); pEGFP-tSSX2 (lane 8, about 38 kDa); pDsRedmonomer-tSSX2 (lane 9, about 42 kDa); and pDsRedmonomer (lane 10); the cell extracts were detected using western blotting with anti-SSX antibody.
(TIF)

Figure S4 **Changes in the localization of SS18-SSX1 when co-expressed with DsRedmonomer tagged truncated SS18 or SSX1 proteins in SYO-1 cells.** A, co-expression of GFP-SS18-SSX1 (A1) and DsRedmonomer-tSS18 (A2); B, co-expression of GFP-SS18-SSX1 (B1) and DsRedmonomer-tSSX1 (B2). Scale bars indicate 5 µm.
(TIF)

Figure S5 **Changes in the localization of SS18-SSX2 when co-expressed with DsRedmonomer-tSS18 or -tSSX2 proteins in HEK293 cells.** pEGFP-SS18-SSX2 (2 µg) was transfected in HEK293 cells with 6 µg of pDsRedmonomer (left), pDsRedmonomer-tSSX2 (middle), and pDsRedmonomer-tSS18 (right). Upper, GFP protein; middle, DsRedmonomer protein; lower, merged image. White arrow shows a cell with speckled pattern of SS18-SSX2 localization in which DsRedmonomer-tSSX2 was not expressed. Scale bars indicate 10 µm.
(TIF)

Figure S6 **Changes in the localization of SS18-SSX when co-expressed with DsRedmonomer tagged truncated SS18, SSX1 or SSX2 proteins in HEK293 cells.** A, co-expression of GFP-SS18-SSX1 (A1) and DsRedmonomer-tSS18 (A2); B, co-expression of GFP-SS18-SSX2 (B1) and DsRedmonomer-tSS18 (B2): C, co-expression of GFP-SS18-SSX1 (C1) and DsRedmonomer-tSSX1 (C2); D, co-expression of GFP-SS18-SSX2 (D1) and DsRedmonomer-tSSX2 (D2). Scale bars indicate 5 µm.
(TIF)

Figure S7 **Effect of DsRedmonomer-tSSX2 and -tSS18 on GFP-SS18-SSX2 expression in transfected HEK293 cells.** Plasmid pEGFP-SS18-SSX2 (2 µg) was transfected into HEK293 cells with 6 µg of pDsRedmonomer (lane 1), pDsRedmonomer-tSSX2 (lane 2), and pDsRedmonomer-tSS18 (lane 3), and the total extracts (10 µg) were analyzed by western blotting with anti-SS18 antibody (upper), anti-FLAG antibody (middle), and anti-β actin antibody (lower). The pDsRedmonomer vector contains the FLAG-tag.
(TIF)

## References

[1] Palmerini E, Staals EL, Alberghini M, Zanella L, Ferrari C et al. (2009) Synovial sarcoma: retrospective analysis of 250 patients treated at a single institution. Cancer 115: 2988-2998. doi:10.1002/cncr.24370. PubMed: 19452538. 19452538

[2] Al-Hussaini H, Hogg D, Blackstein ME, O’Sullivan B, Catton CN et al. (2011) Clinical features, treatment, and outcome in 102 adult and pediatric patients with localized high-grade synovial sarcoma. Sarcoma, 2011: 231789 PubMed: 21559258 10.1155/2011/231789 PMC3087894 21559258

[3] Shi W, Indelicato DJ, Morris CG, Scarborough MT, Gibbs CP et al. (2013) Long-term treatment outcomes for patients with synovial sarcoma: A 40-year experience at the University of Florida. Am J Clin Oncol 36: 83-88. doi:10.1097/COC.0b013e31823fe450. PubMed: 22270107. 22270107

[4] Paulino AC (2004) Synovial sarcoma prognostic factors and patterns of failure. Am J Clin Oncol 27: 122-127. doi:10.1097/01.coc.0000047130.91699.DC. PubMed: 15057149. 15057149

[5] Turc-Carel C, Dal Cin P, Limon J, Li F, Sandberg AA (1986) Translocation X;18 in synovial sarcoma. Cancer Genet Cytogenet 23: 93. doi:10.1016/0165-4608(86)90153-6. PubMed: 3017544. 3017544

[6] Clark J, Rocques PJ, Crew AJ, Gill S, Shipley J et al. (1994) Identification of novel genes, SYT and SSX, involved in the t(X;18)(p11.2;q11.2) translocation found in human synovial sarcoma. Nat Genet 7: 502-508. doi:10.1038/ng0894-502. PubMed: 7951320. 7951320

[7] Crew AJ, Clark J, Fisher C, Gill S, Grimer R et al. (1995) Fusion of SYT to two genes, SSX1 and SSX2, encoding proteins with homology to the Kruppel-associated box in human synovial sarcoma. EMBO J 14: 2333-2340. PubMed: 7539744. 7539744 10.1002/j.1460-2075.1995.tb07228.x PMC398342

[8] dos Santos NR, de Bruijn DR, van Kessel AG (2001) Molecular mechanisms underlying human synovial sarcoma development. Genes Chromosomes Cancer 30: 1-14. doi:10.1002/1098-2264(2000)9999:9999. PubMed: 11107170. 11107170

[9] Perani M, Ingram CJ, Cooper CS, Garrett MD, Goodwin GH (2003) Conserved SNH domain of the proto-oncoprotein SYT interacts with components of the human chromatin remodelling complexes, while the QPGY repeat domain forms homo-oligomers. Oncogene 22: 8156-8167. doi:10.1038/sj.onc.1207031. PubMed: 14603256. 14603256

[10] Thaete C, Brett D, Monaghan P, Whitehouse S, Rennie G et al. (1999) Functional domains of the SYT and SYT-SSX synovial sarcoma translocation proteins and co-localization with the SNF protein BRM in the nucleus. Hum Mol Genet 8: 585-591. doi:10.1093/hmg/8.4.585. PubMed: 10072425. 10072425

[11] Kato H, Tjernberg A, Zhang W, Krutchinsky AN, An W et al. (2002) SYT associates with human SNF/SWI complexes and the C-terminal region of its fusion partner SSX1 targets histones. J Biol Chem 277: 5498-5505. doi:10.1074/jbc.M108702200. PubMed: 11734557. 11734557

[12] Nagai M, Tanaka S, Tsuda M, Endo S, Kato H et al. (2001) Analysis of transforming activity of human synovial sarcoma-associated chimeric protein SYT-SSX1 bound to chromatin remodeling factor hBRM/hSNF2 alpha. Proc Natl Acad Sci U S A 98: 3843-3848. doi:10.1073/pnas.061036798. PubMed: 11274403. 11274403 PMC31140

[13] Ishida M, Tanaka S, Ohki M, Ohta T (2004) Transcriptional co-activator activity of SYT is negatively regulated by BRM and Brg1. Genes Cells 9: 419-428. doi:10.1111/j.1356-9597.2004.00737.x. PubMed: 15147271. 15147271

[14] Ito T, Ouchida M, Ito S, Jitsumori Y, Morimoto Y et al. (2004) SYT, a partner of SYT-SSX oncoprotein in synovial sarcomas, interacts with mSin3A, a component of histone deacetylase complex. Lab Invest 84: 1484-1490. doi:10.1038/labinvest.3700174. PubMed: 15467731. 15467731

[15] Ito T, Ouchida M, Morimoto Y, Yoshida A, Jitsumori Y et al. (2005) Significant growth suppression of synovial sarcomas by the histone deacetylase inhibitor FK228 in vitro and in vivo. Cancer Lett 224: 311-319. doi:10.1016/j.canlet.2004.10.030. PubMed: 15914281. 15914281

[16] Eid JE, Kung AL, Scully R, Livingston DM (2000) p300 interacts with the nuclear proto-oncoprotein SYT as part of the active control of cell adhesion. Cell 102: 839-848. doi:10.1016/S0092-8674(00)00072-6. PubMed: 11030627. 11030627

[17] de Bruijn DR, dos Santos NR, Thijssen J, Balemans M, Debernardi S et al. (2001) The synovial sarcoma associated protein SYT interacts with the acute leukemia associated protein AF10. Oncogene 20: 3281-3289. doi:10.1038/sj.onc.1204419. PubMed: 11423977. 11423977

[18] Naka N, Joyama S, Tsukamoto Y, Yoshioka K, Hashimoto N et al. (2005) Quantification of SSX mRNA expression in human bone and soft tissue tumors using nucleic acid sequence-based amplification. J Mol Diagn 7: 187-197. doi:10.1016/S1525-1578(10)60545-4. PubMed: 15858142. 15858142 PMC1867521

[19] Türeci O, Sahin U, Schobert I, Koslowski M, Scmitt H et al. (1996) The SSX-2 gene, which is involved in the t(X;18) translocation of synovial sarcomas, codes for the human tumor antigen HOM-MEL-40. Cancer Res 56: 4766-4772. PubMed: 8840996. 8840996

[20] Gure AO, Türeci O, Sahin U, Tsang S, Scanlan MJ et al. (1997) SSX: a multigene family with several members transcribed in normal testis and human cancer. Int J Cancer 72: 965-971. doi:10.1002/(SICI)1097-0215(19970917)72:6. PubMed: 9378559. 9378559

[21] Türeci O, Chen YT, Sahin U, Güre AO, Zwick C et al. (1998) Expression of SSX genes in human tumors. Int J Cancer 77: 19-23. doi:10.1002/(SICI)1097-0215(19980703)77:1. PubMed: 9639388. 9639388

[22] Skytting B, Nilsson G, Brodin B, Xie Y, Lundeberg J et al. (1999) A novel fusion gene, SYT-SSX4, in synovial sarcoma. J Natl Cancer Inst 91: 974-975. doi:10.1093/jnci/91.11.974. PubMed: 10359553. 10359553

[23] Brodin B, Haslam K, Yang K, Bartolazzi A, Xie Y et al. (2001) Cloning and characterization of spliced fusion transcript variants of synovial sarcoma: SYT/SSX4, SYT/SSX4v, and SYT/SSX2v. Possible regulatory role of the fusion gene product in wild-type SYT expression. Gene 268: 173-182. doi:10.1016/S0378-1119(01)00412-7. PubMed: 11368913. 11368913

[24] Güre AO, Wei IJ, Old LJ, Chen YT (2002) The SSX gene family: characterization of 9 complete genes. Int J Cancer 101: 448-453. doi:10.1002/ijc.10634. PubMed: 12216073. 12216073

[25] dos Santos NR, de Bruijn DR, Kater-Baats E, Otte AP, van Kessel AG (2000) Delineation of the protein domains responsible for SYT, SSX, and SYT-SSX nuclear localization. Exp Cell Res 256: 192-202. doi:10.1006/excr.2000.4813. PubMed: 10739666. 10739666

[26] Soulez M, Saurin AJ, Freemont PS, Knight JC (1999) SSX and the synovial-sarcoma-specific chimaeric protein SYT-SSX co-localize with the human Polycomb group complex. Oncogene 18: 2739-2746. doi:10.1038/sj.onc.1202613. PubMed: 10348348. 10348348

[27] Smith HA, McNeel DG (2010) The SSX family of cancer-testis antigens as target proteins for tumor therapy. Clin Dev Immunol, 2010: 150591 PubMed: 20981248 10.1155/2010/150591 PMC2963798 20981248

[28] Przybyl J, Jurkowska M, Rutkowski P, Debiec-Rychter M, Siedlecki JA (2012) Downstream and intermediate interactions of synovial sarcoma-associated fusion oncoproteins and their implication for targeted therapy. Sarcoma, 2012: 249219 PubMed: 22550415 10.1155/2012/249219 PMC3329658 22550415

[29] Barco R, Garcia CB, Eid JE (2009) The synovial sarcoma-associated SYT-SSX2 oncogene antagonizes the polycomb complex protein Bmi1. PLOS ONE 4: e5060. doi:10.1371/journal.pone.0005060. PubMed: 19337376. 19337376 PMC2659801

[30] de Bruijn DR, dos Santos NR, Kater-Baats E, Thijssen J, van den Berk L et al. (2002) The cancer-related protein SSX2 interacts with the human homologue of a Ras-like GTPase interactor, RAB3IP, and a novel nuclear protein, SSX2IP. Genes Chromosomes Cancer 34: 285-298. doi:10.1002/gcc.10073. PubMed: 12007189. 12007189

[31] dos Santos NR, de Bruijn DR, Balemans M, Janssen B, Gärtner F et al. (1997) Nuclear localization of SYT, SSX and the synovial sarcoma-associated SYT-SSX fusion proteins. Hum Mol Genet 6: 1549-1558. doi:10.1093/hmg/6.9.1549. PubMed: 9285793. 9285793

[32] Brett D, Whitehouse S, Antonson P, Shipley J, Cooper C et al. (1997) The SYT protein involved in the t(X;18) synovial sarcoma translocation is a transcriptional activator localised in nuclear bodies. Hum Mol Genet 6: 1559-1564. doi:10.1093/hmg/6.9.1559. PubMed: 9285794. 9285794

[33] Saito T, Nagai M, Ladanyi M (2006) SYT-SSX1 and SYT-SSX2 interfere with repression of E-cadherin by snail and slug: a potential mechanism for aberrant mesenchymal to epithelial transition in human synovial sarcoma. Cancer Res 66: 6919-6927. doi:10.1158/0008-5472.CAN-05-3697. PubMed: 16849535. 16849535

[34] Xie Y, Törnkvist M, Aalto Y, Nilsson G, Girnita L et al. (2003) Gene expression profile by blocking the SYT-SSX fusion gene in synovial sarcoma cells. Identification of XRCC4 as a putative SYT-SSX target gene. Oncogene 22: 7628-7631. doi:10.1038/sj.onc.1207153. PubMed: 14576825. 14576825

[35] Tsuda M, Watanabe T, Seki T, Kimura T, Sawa H et al. (2005) Induction of p21 (WAF1/CIP1) by human synovial sarcoma-associated chimeric oncoprotein SYT-SSX1. Oncogene 24: 7984-7990. doi:10.1038/sj.onc.1208942. PubMed: 16103879. 16103879

[36] Fernebro J, Francis P, Edén P, Borg A, Panagopoulos I et al. (2006) Gene expression profiles relate to SS18/SSX fusion type in synovial sarcoma. Int J Cancer 118: 1165-1172. doi:10.1002/ijc.21475. PubMed: 16152617. 16152617

[37] Horvai AE, Kramer MJ, O’Donnell R (2006) Beta-catenin nuclear expression correlates with cyclin D1 expression in primary and metastatic synovial sarcoma: a tissue microarray study. Arch Pathol Lab Med 130: 792-798. PubMed: 16740029. 16740029 10.5858/2006-130-792-CNECWC

[38] Sun Y, Gao D, Liu Y, Huang J, Lessnick S et al. (2006) IGF2 is critical for tumorigenesis by synovial sarcoma oncoprotein SYT-SSX1. Oncogene 25: 1042-1052. doi:10.1038/sj.onc.1209143. PubMed: 16247461. 16247461

[39] de Bruijn DR, Allander SV, van Dijk AH, Willemse MP, Thijssen J et al. (2006) The synovial-sarcoma-associated SS18-SSX2 fusion protein induces epigenetic gene (de)regulation. Cancer Res 66: 9474-9482. doi:10.1158/0008-5472.CAN-05-3726. PubMed: 17018603. 17018603

[40] de Bruijn DR, Nap JP, van Kessel AG (2007) The (epi)genetics of human synovial sarcoma. Genes Chromosomes Cancer 46: 107-117. doi:10.1002/gcc.20399. PubMed: 17117414. 17117414

[41] Ishida M, Miyamoto M, Naitoh S, Tatsuda D, Hasegawa T et al. (2007) The SYT-SSX fusion protein down-regulates the cell proliferation regulator COM1 in t(x;18) synovial sarcoma. Mol Cell Biol 27: 1348-1355. doi:10.1128/MCB.00658-06. PubMed: 17101797. 17101797 PMC1800732

[42] Törnkvist M, Natalishvili N, Xie Y, Girnita A, D’Arcy P et al. (2008) Differential roles of SS18-SSX fusion gene and insulin-like growth factor-1 receptor in synovial sarcoma cell growth. Biochem Biophys Res Commun 368: 793-800. doi:10.1016/j.bbrc.2008.01.162. PubMed: 18267106. 18267106

[43] Lubieniecka JM, de Bruijn DR, Su L, van Dijk AH, Subramanian S et al. (2008) Histone deacetylase inhibitors reverse SS18-SSX-mediated polycomb silencing of the tumor suppressor early growth response 1 in synovial sarcoma. Cancer Res 68: 4303–4310. doi:10.1158/0008-5472.CAN-08-0092. PubMed: 18519690. 18519690

[44] Su L, Sampaio AV, Jones KB, Pacheco M, Goytain A et al. (2012) Deconstruction of the SS18-SSX fusion oncoprotein complex: insights into disease etiology and therapeutics. Cancer Cell 21: 333-347. doi:10.1016/j.ccr.2012.01.010. PubMed: 22439931. 22439931 PMC3734954

[45] Naka N, Takenaka S, Araki N, Miwa T, Hashimoto N et al. (2010) Synovial sarcoma is a stem cell malignancy. Stem Cells 28: 1119–1131. PubMed: 20518020. 20518020 10.1002/stem.452

[46] Garcia CB, Shaffer CM, Alfaro MP, Smith AL, Sun J et al. (2012) Reprogramming of mesenchymal stem cells by the synovial sarcoma-associated oncogene SYT-SSX2. Oncogene 31: 2323–2334. doi:10.1038/onc.2011.418. PubMed: 21996728. 21996728 PMC3752676

[47] Wilson BG, Wang X, Shen X, McKenna ES, Lemieux ME et al. (2010) Epige- netic antagonism between polycomb and SWI/SNF complexes during onco- genic transformation. Cancer Cell 18: 316–328. doi:10.1016/j.ccr.2010.09.006. PubMed: 20951942. 20951942 PMC2957473

[48] Kadoch C, Crabtree GR (2013) Reversible disruption of mSWI/SNF (BAF) complexes by the SS18-SSX oncogenic fusion in synovial sarcoma. Cell 153: 71-85. doi:10.1016/j.cell.2013.02.036. PubMed: 23540691. 23540691 PMC3655887

[49] Haldar M, Hancock JD, Coffin CM, Lessnick SL, Capecchi MR (2007) A conditional mouse model of synovial sarcoma: insights into a myogenic origin. Cancer Cell 11: 375–388. doi:10.1016/j.ccr.2007.01.016. PubMed: 17418413. 17418413

[50] Kawai A, Naito N, Yoshida A, Morimoto Y, Ouchida M et al. (2004) Establishment and characterization of a biphasic synovial sarcoma cell line, SYO-1. Cancer Lett 204: 105-113. doi:10.1016/j.canlet.2003.09.031. PubMed: 14744540. 14744540

[51] Lim FL, Soulez M, Koczan D, Thiesen HJ, Knight JC (1998) A KRAB-related domain and a novel transcription repression domain in proteins encoded by SSX genes that are disrupted in human sarcomas. Oncogene 17: 2013-2018. doi:10.1038/sj.onc.1202122. PubMed: 9788446. 9788446

[52] Ishibe T, Nakayama T, Okamoto T, Aoyama T, Nishijo K et al. (2005) Disruption of fibroblast growth factor signal pathway inhibits the growth of synovial sarcomas: potential application of signal inhibitors to molecular target therapy. Clin Cancer Res 11(7): 2702-2712. doi:10.1158/1078-0432.CCR-04-2057. PubMed: 15814652. 15814652

[53] Morimoto Y, Ouchida M, Ozaki T, Kawai A, Ito T et al. (2003) Splicing isoform of SYT-SSX fusion protein accelerates transcriptional activity and cell proliferation. Cancer Lett 199(1): 35-43. doi:10.1016/S0304-3835(03)00314-8. PubMed: 12963121. 12963121

