# Supplementary figures and images for: Truncated SSX Protein Suppresses Synovial Sarcoma Cell Proliferation by Inhibiting the Localization of SS18-SSX Fusion Protein
Source: PLoS One. 2013 Oct 9;8(10):e77564. doi: 10.1371/journal.pone.0077564 (PMC3793959; doi:10.1371/journal.pone.0077564)

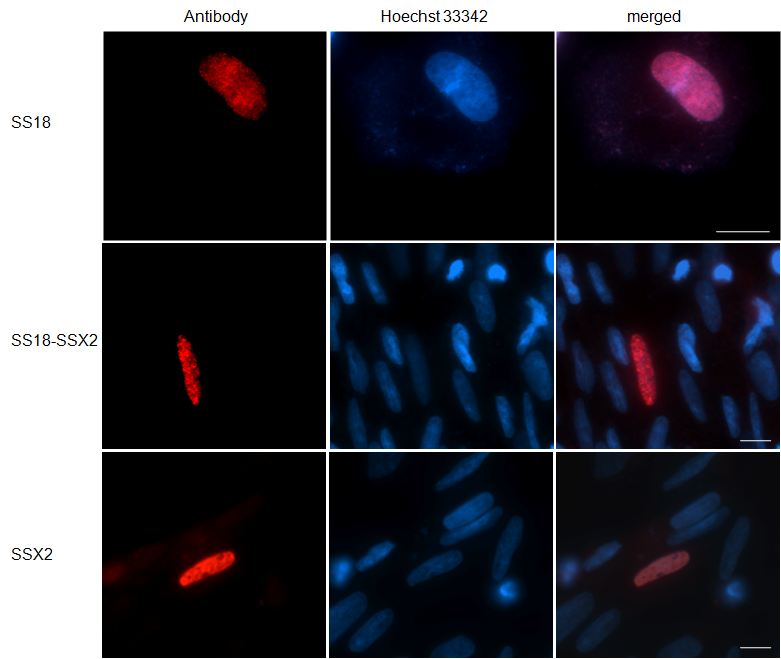

Supplement: Figure S1 — Localization of synovial sarcoma-related proteins by fluorescence immunocytochemistry. HEK293 cells were transfected with pCMV-Tag2B-SS18, pCMV-Tag2B-SSX2, and pCMV-Tag2B-SS18-SSX2, and analyzed by fluorescence immunocytochemistry with anti-SS18 and anti-SSX antibodies. The transfected cells with SS18 and SS18-SSX2 were reacted with anti-SS18 antibody (upper and middle, respectively), and the SSX2 transfectant was reacted with anti-SSX antibody (lower). Left, antibody reaction using Alexa 594-conjugated secondary antibody; middle, Hoechst33342 staining; right, merged image. The scale bars are 5-µm long. (TIF) [file pone.0077564.s001.tif]

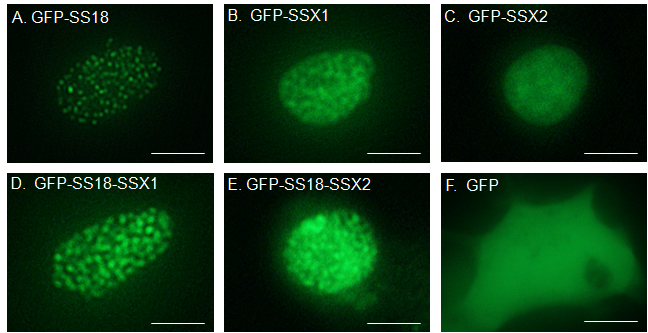

Supplement: Figure S2 — Localization of synovial sarcoma-related fusion proteins in HEK293 cells. Cells expressing GFP-tagged proteins were observed under a fluorescence microscope. A, GFP-SS18; B, GFP-SSX1; C, GFP-SSX2; D, GFP-SS18-SSX1; E, GFP-SS18-SSX2; F, GFP. Scale bars indicate 5 µm. (TIF) [file pone.0077564.s002.tif]

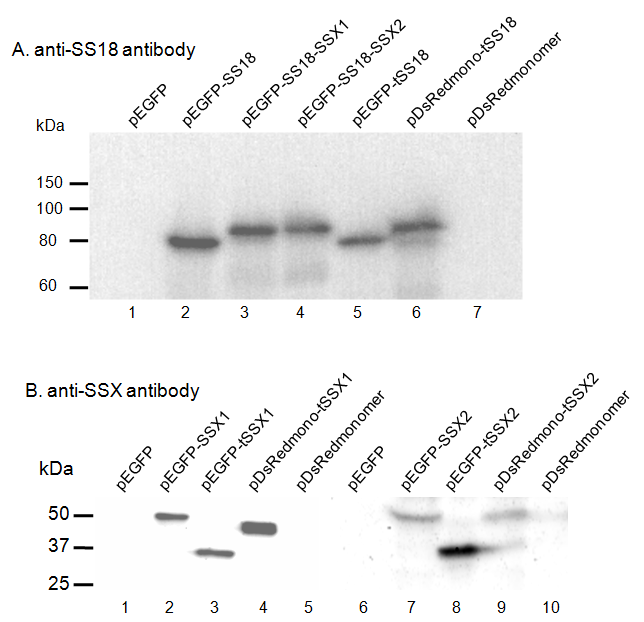

Supplement: Figure S3 — Confirmation of expression of recombinant proteins by Western blotting. A: HEK293 cells were transfected with pEGFP (lane 1); pEGFP-SS18 (lane 2, about 79 kDa); pEGFP-SS18-SSX1 (lane 3, about 83 kDa); pEGFP-SS18-SSX2 (lane 4, about 83 kDa); pEGFP-tSS18 (lane 5, about 77 kDa); pDsRedmonomer-tSS18 (lane 6, about 81 kDa); and pDsRedmonomer (lane 7); and the cell extracts were detected by western blotting with anti-SS18 antibody. B: HEK293 cells were transfected with pEGFP (lane 1); pEGFP-SSX1 (lane 2, about 51 kDa); pEGFP-tSSX1 (lane 3, about 38 kDa); pDsRedmonomer-tSSX1 (lane 4, about 42 kDa); pDsRedmonomer (lane 5); pEGFP (lane 6); pEGFP-SSX2 (lane 7, about 51 kDa); pEGFP-tSSX2 (lane 8, about 38 kDa); pDsRedmonomer-tSSX2 (lane 9, about 42 kDa); and pDsRedmonomer (lane 10); the cell extracts were detected using western blotting with anti-SSX antibody. (TIF) [file pone.0077564.s003.tif]

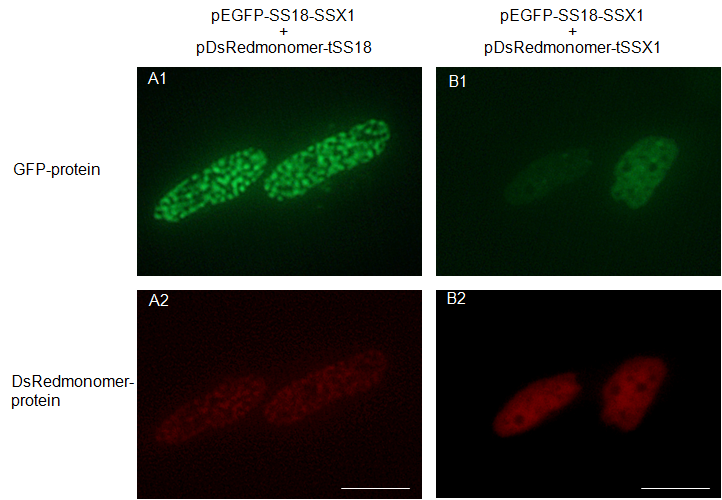

Supplement: Figure S4 — Changes in the localization of SS18-SSX1 when co-expressed with DsRedmonomer tagged truncated SS18 or SSX1 proteins in SYO-1 cells. A, co-expression of GFP-SS18-SSX1 (A1) and DsRedmonomer-tSS18 (A2); B, co-expression of GFP-SS18-SSX1 (B1) and DsRedmonomer-tSSX1 (B2). Scale bars indicate 5 µm. (TIF) [file pone.0077564.s004.tif]

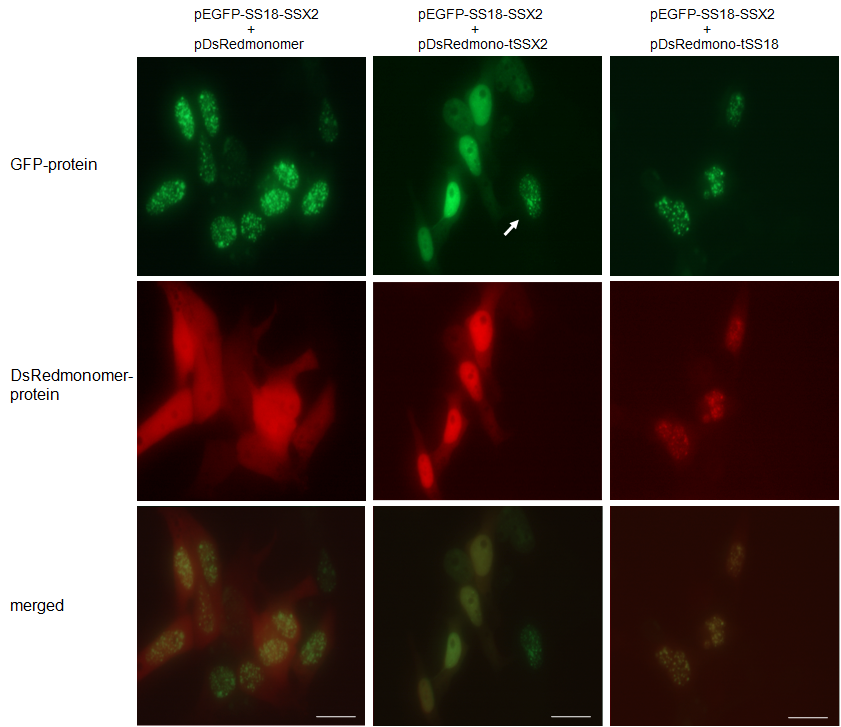

Supplement: Figure S5 — Changes in the localization of SS18-SSX2 when co-expressed with DsRedmonomer-tSS18 or -tSSX2 proteins in HEK293 cells. pEGFP-SS18-SSX2 (2 µg) was transfected in HEK293 cells with 6 µg of pDsRedmonomer (left), pDsRedmonomer-tSSX2 (middle), and pDsRedmonomer-tSS18 (right). Upper, GFP protein; middle, DsRedmonomer protein; lower, merged image. White arrow shows a cell with speckled pattern of SS18-SSX2 localization in which DsRedmonomer-tSSX2 was not expressed. Scale bars indicate 10 µm. (TIF) [file pone.0077564.s005.tif]

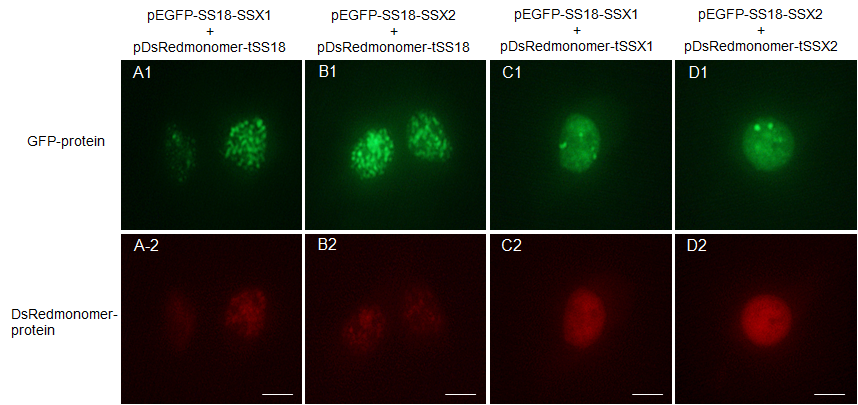

Supplement: Figure S6 — Changes in the localization of SS18-SSX when co-expressed with DsRedmonomer tagged truncated SS18, SSX1 or SSX2 proteins in HEK293 cells. A, co-expression of GFP-SS18-SSX1 (A1) and DsRedmonomer-tSS18 (A2); B, co-expression of GFP-SS18-SSX2 (B1) and DsRedmonomer-tSS18 (B2): C, co-expression of GFP-SS18-SSX1 (C1) and DsRedmonomer-tSSX1 (C2); D, co-expression of GFP-SS18-SSX2 (D1) and DsRedmonomer-tSSX2 (D2). Scale bars indicate 5 µm. (TIF) [file pone.0077564.s006.tif]

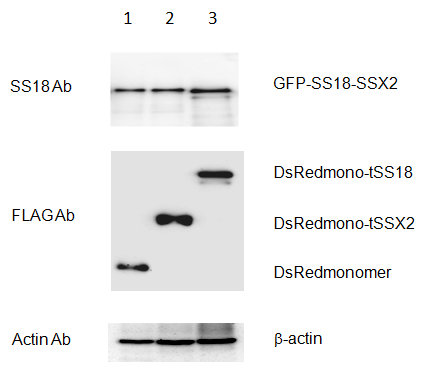

Supplement: Figure S7 — Effect of DsRedmonomer-tSSX2 and -tSS18 on GFP-SS18-SSX2 expression in transfected HEK293 cells. Plasmid pEGFP-SS18-SSX2 (2 µg) was transfected into HEK293 cells with 6 µg of pDsRedmonomer (lane 1), pDsRedmonomer-tSSX2 (lane 2), and pDsRedmonomer-tSS18 (lane 3), and the total extracts (10 µg) were analyzed by western blotting with anti-SS18 antibody (upper), anti-FLAG antibody (middle), and anti-β actin antibody (lower). The pDsRedmonomer vector contains the FLAG-tag. (TIF) [file pone.0077564.s007.tif]
